# Role of traditional Chinese medicine in ameliorating mitochondrial dysfunction *via* non-coding RNA signaling: Implication in the treatment of neurodegenerative diseases

**DOI:** 10.3389/fphar.2023.1123188

**Published:** 2023-03-01

**Authors:** Zhongdi Cai, Mimin Liu, Li Zeng, Kaiyue Zhao, Chenyu Wang, Ting Sun, Zhuorong Li, Rui Liu

**Affiliations:** Institute of Medicinal Biotechnology, Chinese Academy of Medical Sciences and Peking Union Medical College, Beijing, China

**Keywords:** mitochondrial dysfunction, multi-targeting, neurodegenerative diseases, non-coding RNAs, traditional Chinese medicine

## Abstract

Neurodegenerative diseases (NDs) are common chronic disorders associated with progressive nervous system damage, including Alzheimer’s disease, Parkinson’s disease, and Huntington’s disease, among others. Mitochondria are abundant in various nervous system cells and provide a bulk supply of the adenosine triphosphate necessary for brain function, considered the center of the free-radical theory of aging. One common feature of NDs is mitochondrial dysfunction, which is involved in many physiopathological processes, including apoptosis, inflammation, oxidative stress, and calcium homeostasis. Recently, genetic studies revealed extensive links between mitochondrion impairment and dysregulation of non-coding RNAs (ncRNAs) in the pathology of NDs. Traditional Chinese medicines (TCMs) have been used for thousands of years in treating NDs. Numerous modern pharmacological studies have demonstrated the therapeutic effects of prescription, herbal medicine, bioactive ingredients, and monomer compounds of TCMs, which are important for managing the symptoms of NDs. Some highly effective TCMs exert protective effects on various key pathological features regulated by mitochondria and play a pivotal role in recovering disrupted signaling pathways. These disrupted signaling pathways are induced by abnormally-expressed ncRNAs associated with mitochondrial dysfunction, including microRNAs, long ncRNAs, and circular RNAs. In this review, we first explored the underlying ncRNA mechanisms linking mitochondrial dysfunction and neurodegeneration, demonstrating the implication of ncRNA-induced mitochondrial dysfunction in the pathogenesis of NDs. The ncRNA-induced mitochondrial dysfunctions affect mitochondrial biogenesis, dynamics, autophagy, Ca^2+^ homeostasis, oxidative stress, and downstream apoptosis. The review also discussed the targeting of the disease-related mitochondrial proteins in NDs and the protective effects of TCM formulas with definite composition, standardized extracts from individual TCMs, and monomeric compounds isolated from TCM. Additionally, we explored the ncRNA regulation of mitochondrial dysfunction in NDs and the effects and potential mechanisms of representative TCMs in alleviating mitochondrial pathogenesis and conferring anti-inflammatory, antioxidant, and anti-apoptotic pathways against NDs. Therefore, this review presents an overview of the role of mitochondrion-related ncRNAs and the target genes for TCM-based therapeutic interventions in NDs, providing insight into understanding the “multi-level compound-target-pathway regulatory” treatment mechanism of TCMs.

## GRAPHICAL ABSTRACT

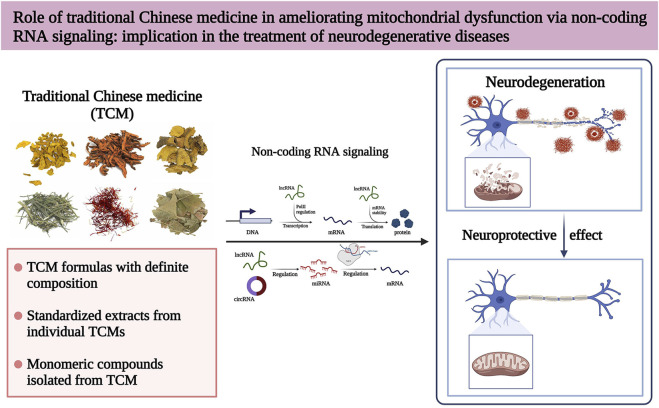

## 1 Introduction

Neurodegenerative diseases (NDs) are a set of disorders with intricate etiologies manifesting as heterogeneous symptoms which impact different areas of the brain and spinal cord. Mitochondrial dysfunction is a major pathogenic factor in the occurrence and development of NDs associated with aging, such as Alzheimer’s disease (AD), Parkinson’s disease (PD), Amyotrophic lateral sclerosis (ALS), and Huntington’s disease (HD). When the brain ages, its metabolic rate decreases, resulting in pathological features such as mitochondrial malfunction, abnormal energy metabolism, calcium imbalance, cell cycle deregulation, apoptosis, and reactive oxygen species (ROS) production. Mitochondria are the synthesis site of 90% of adenosine triphosphate (ATP) and the power source which maintains various vital activities in the body. Several studies reported that many patients with NDs exhibit abnormal glucose metabolism in the cerebral cortex and hippocampus, which damages the oxidative phosphorylation system, before developing obvious clinicopathological symptoms, such as neuron loss and cognitive decline (Jadiya et al., 2021). This suggested that mitochondrial dysfunction may be an early sign of NDs. Therefore, mitochondrial impairments are regarded as common neurodegeneration signatures during aging.

Mitochondria are involved in various cellular activities, including ROS production, ionic homeostasis maintenance, fatty acid decomposition, and apoptosis regulation. Alterations in mitochondrial motility, biogenesis, morphology, dynamics, or mutations of the mitochondrial DNA cause variation in nuclear-encoded mitochondrial genes, affecting normal mitochondrial functions *via* anterograde signalings. Mitochondrial dysfunction can cause neurodegeneration through various mechanisms, including interference with cell signaling pathways, oxidative stress, apoptosis, and microglia activation (Johnson et al., 2021). Neurodegeneration can also exacerbate mitochondrial dysfunction and further aggravate NDs, creating a vicious cycle. However, the detailed molecular mechanisms by which mitochondrial dysfunction affects neurodegeneration remain largely unknown. Recently, there have been increasing reports that non-coding RNAs (ncRNAs) and various endogenous regulators, including microRNAs (miRNAs), long ncRNAs (lncRNAs), and circular RNAs (circRNAs), are implicated in mitochondrial dysfunction and mitochondrion associated signaling transduction (du Mee et al., 2020; Zeng et al., 2021; Sun et al., 2022a; Jiang et al., 2022). A small proportion of ncRNAs found in mitochondria are reportedly transcribed from the mitochondrial genome, while the majority are nuclear-encoded ncRNAs that enter the mitochondria from the nucleus (Sharma et al., 2019). These ncRNAs directly regulate mitochondrial gene expression or indirectly act on different signaling pathways resulting in mitochondrial dysfunction. Therefore, ncRNAs have been demonstrated to play an important role in the etiology of NDs by mediating neurodegeneration *via* mitochondrial dysfunction.

**FIGURE 1:**
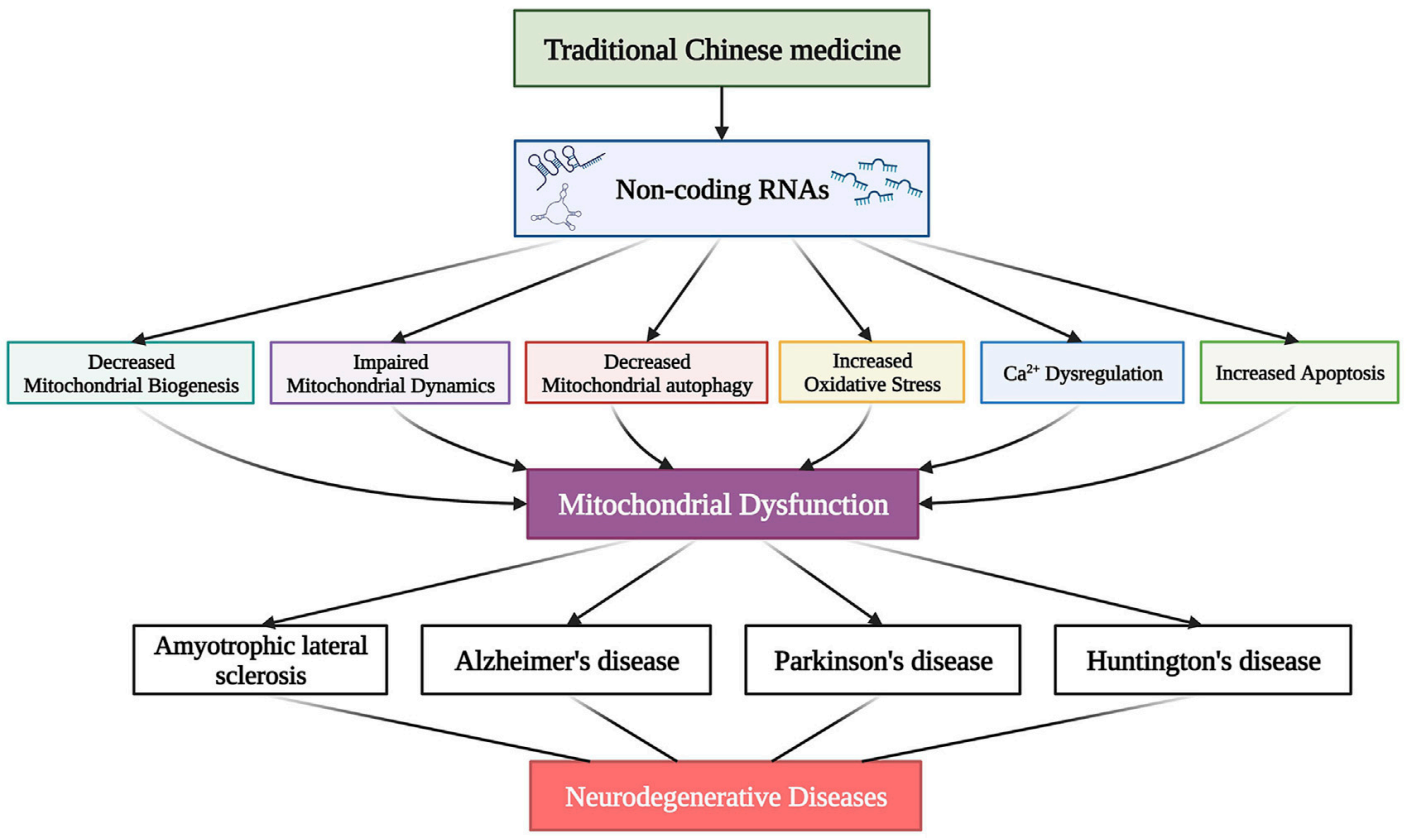
Traditional Chinese medicine (TCM) modulates mitochondrial dysfunction by directly acting on ncRNAs to alleviate various neurodegenerative diseases (NDs).

Traditional Chinese medicine (TCM) has become one of the most important sources for exploring and developing modern medicines due to their application in treating various diseases for more than 5,000 years (Wang et al., 2018). Additionally, TCM can be further optimized to treat complex diseases due to its multi-component, multi-target, and multi-pathway synergistic effects (Liu et al., 2021a; Cai et al., 2022a). Over the past few years, TCM has received increasing attention in treating NDs, and many NDs treatments of TCM origin are in different clinical trial stages (Shan et al., 2018). In this review, we discuss the mitochondrial dysfunction regulatory mechanisms of ncRNAs implicated in NDs and summarize various TCMs that treat NDs by regulating mitochondrial dysfunction from modern pharmacological studies. We also highlight the possibility that some TCMs can treat NDs by interfering with ncRNAs to regulate mitochondrial dysfunction (Figure 1). Thus, this review emphasizes the broad application prospects of TCM, aiming to provide new insights and strategies for treating NDs.

## 2 ncRNAs target mitochondria to regulate NDs

**FIGURE 2:**
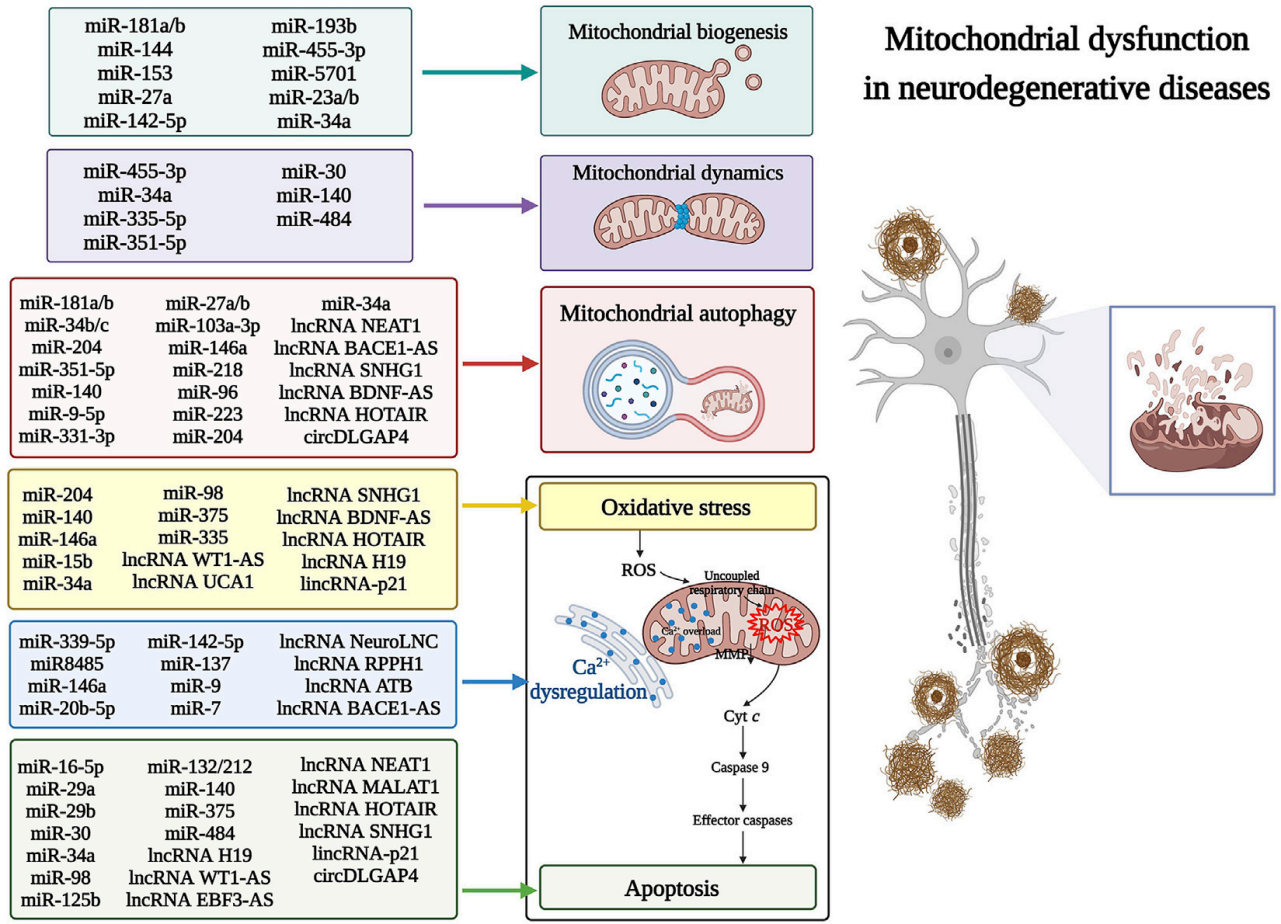
The non-coding RNAs (ncRNAs) that regulate mitochondrial dysfunction in neurodegenerative diseases (NDs). Various mitochondrial dysfunctions are associated with the dysregulation of ncRNAs in NDs. These dysfunctions include the pathogenesis of mitochondrial biogenesis, dynamics, autophagy, calcium dyshomeostasis, reactive oxygen species (ROS)-mediated mitochondrial oxidative stress, and downstream apoptosis. Cyt *c*, cytochrome *c*; ROS, reactive oxygen species; MMP, mitochondrial membrane potential.

Mitochondrial dysfunction, such as reduced mitochondrial biogenesis, impaired mitochondrial dynamics, abnormal mitochondrial autophagy (mitophagy), Ca^2+^ overload, activation of ROS-mediated oxidative stress, and downstream apoptosis, are the main research areas in NDs. Mitochondria are susceptible to various genetic and environmental factors. ncRNAs regulate the intersection of mitochondrial signaling pathways by modulating the levels of transcriptional and post-transcriptional mitochondrion-related genes. Therefore, identifying ncRNA-mediated mitochondrion-targeting drugs may provide new therapeutic targets for NDs. Figure 2 highlights the mitochondrial dysfunctions during NDs and summarizes the corresponding dysfunction of ncRNAs.

### 2.1 ncRNAs regulate mitochondrial gene expression

Regulation of mitochondrial biogenesis and dynamics, accompanied by mitochondrial clearance and quality control, has been used as a possible treatment strategy for mitochondrial diseases. microRNA-181a and 181b (miR-181a/b), belonging to the miR-181 family, have been highly expressed in various brain regions of AD patients. Additionally, miR-181a/b has been recently shown to target key genes regulating mitochondrial biogenesis and mitophagy. These genes included PPARG coactivator 1-alpha (PPARGC1A), nuclear respiratory factor 1 (NRF1) (major regulators of mitochondrial biogenesis), cytochrome *c* oxidase copper chaperone COX11 (COX11), coenzyme Q10B (COQ10B) (involved in mitochondrial respiratory chain assembly), and autophagy-related 5 (ATG5) and parkin RBR E3 ubiquitin-protein ligase (PARK2) (key players in mitophagy). Knockdown of miR-181a/b enhanced the expression of mitochondrial biogenesis-related genes and mitophagy protein markers, maintaining mitochondrial homeostasis and alleviating neural deformation (Indrieri et al., 2019; Figure 2).

The stability of the internal mitochondrial metabolism is regulated by the dynamic processes of mitochondrial fusion and fission. A recent study found that miR-351-5p directly targeted mitochondrial Rho GTPase 2 (MIRO2), inhibiting its expression and causing large mitochondrial fission and fragmentation, thereby leading to neural progenitor cell death in the hippocampus (Woo et al., 2020; Figure 2). Furthermore, such miRNA regulation is reportedly associated with a functional correlation between mitochondrial dynamics and mitophagy. Excessive mitochondrial fission promotes mitophagy through the PTEN-induced kinase 1 (PINK1)/Parkin-mediated mitophagy pathway, with a convergence of the miR-351-5p/MIRO2 axis in the pathology of AD (Chen et al., 2016). With the ongoing development of high-throughput sequencing and microarray technologies, many novel ncRNAs with regulatory roles in NDs are being identified (Cai et al., 2022b; Sun et al., 2022b; Zhao et al., 2022). For example, miR-455-3p is a newly discovered miRNA that showed significant up-regulation in AD (Kumar et al., 2017), and its overexpression positively regulated the expression of mitochondrial biogenesis genes. This increased the mRNA and protein levels of key genes [PPARGC1A, NRF1, NRF2, transcription factor A, and mitochondrial (TFAM)] in mitochondrial biogenesis. Moreover, miR-455-3p decreased the mitochondrial fission proteins [Dynamin-related protein 1 (DRP1) and mitochondrial fission 1 (FIS1)] but significantly increased the fusion proteins [Optic nerve atrophy 1 (OPA1), Mitogen 1 (MFN1), and 2 (MFN2)], protecting the mitochondria from the toxic effects of β-amyloid (Aβ) (Kumar et al., 2019; Figure 2).

### 2.2 ncRNAs regulate Ca^2+^ homeostasis, ROS-mediated oxidative stress, and downstream apoptotic machinery in mitochondria

In addition to their role in bioenergy production, mitochondria display other important cellular functions, including regulating Ca^2+^ homeostasis, ROS signaling, and apoptosis. Intracytoplasmic Ca^2+^ homeostasis is essential for neurons to conduct and sustain their physiological activities. Mitochondria are calcium buffering sites that protect cells against high cytoplasmic Ca^2+^. However, impaired synaptic function and synaptic loss cause a continuous increase of Ca^2+^ concentration in the neuron cytoplasm, leading to mitochondrial oxidative stress, membrane potential changes, reduced ATP production, and ultimately affecting mitochondrial function. The highly neuro-specific lncRNA, NeuroLNC, affected calcium dynamics by interacting with the RNA-binding protein TAR DNA binding protein (TARDBP), which promotes selective stabilization of presynaptic protein-encoded mRNA (Keihani et al., 2019; Figure 2). Moreover, mutations in miR8485, another ncRNA that binds TARDBP, led to overexpression of neurexin 1 (NRXN1), resulting in presynaptic Ca^2+^ overload, mitochondrial Ca^2+^ uptake, and neurodegeneration (Fan et al., 2014; Kattimani and Veerappa, 2018). Under pathological conditions of NDs, intramitochondrial calcium overload, caused by intracellular calcium imbalance, promotes ROS production from different sources and induces downstream apoptosis by complementary action of ROS overexposure (Baev et al., 2022; Figure 2).

In addition to directly regulating mitochondrial genes, some ncRNAs, such as miR-34a and miR-7, can also regulate mitochondrial oxidative stress and downstream apoptosis to influence the onset and progression of NDs (Figure 2). miR-34a, a miRNA specifically up-regulated in AD and used in distinguishing AD patients from those with PD or HD, disrupts mitochondrial oxidative phosphorylation by inhibiting the expression of electron transport chain components (Sarkar et al., 2016). Moreover, overexpressing miR-34a reduced the *PPARGC1A*, *NRF1*, and *TFAM* levels significantly, inhibiting mitochondrial biogenesis (Thounaojam et al., 2019). Knockdown of miR-34a increased the levels of autophagy-related proteins, such as microtubule-associated protein one light chain three alpha (LC3) II/I, beclin 1 (BECN1), and ATG7, accelerated degradation of P62, and induced abnormal expression of DRP1 and MFN2. This improved autophagy and mitochondrial dynamics (Kou et al., 2017). In addition, miR-34a was reportedly up-regulated in the frontal cortex and hippocampus of early AD patients in the BraaK phase III/IV and was shown to be embedded in an upstream regulator of oxidative stress (Nunomura and Perry, 2020). Furthermore, targeting the anti-apoptotic protein BCL2 apoptosis regulator (BCL2) with miR-34a inhibited endogenous apoptosis and promoted neuroprotection (Cosín-Tomás et al., 2017). The multi-target effects of miR-34a reported on oxidative stress, mitochondrial health, and apoptosis in AD are due to its multi-functional activities in mitochondria. miR-7 significantly reduced the brain regions associated with dopaminergic neurodegeneration among PD patients. Currently, miR-7 replacement therapy has been proposed to slow PD progression from the early stages by modulating mitochondrial function, apoptosis, oxidative stress, and directly targeting PD-related genes. This reduces α-synuclein (α-Syn) accumulation in Lewy bodies and enhances the survival of the remaining neurons, indicating the potential for key mechanisms involved in neuropathology (Titze-de-Almeida and Titze-de-Almeida, 2018). Recent studies have shown that lncRNA small molecule RNA host gene 1 (SNHG1) up-regulated in PD can regulate the expression of BCL2 associated X, an apoptosis regulator (BAX), by interacting with miR-216a-3p (Wang et al., 2021a). SNHG1 can also regulate apoptosis in PD by binding miR-153-3p to modulate PTEN/Akt/mTOR signaling (Zhao et al., 2020). Moreover, silencing SNHG1 reportedly protected SK-N-SH and MN9D cells from 1-methyl-4-phenylpyridinium (MPP^+^)-induced oxidative stress (Xiao et al., 2021) and promoted mitophagy (Qian et al., 2019). These multi-linked activities make SNHG1 a potential therapeutic target for treating PD (Figure 2). So far, compared to miRNAs and lncRNAs, there are fewer studies on the involvement of circRNAs in NDs. CircDLGAP4 is reportedly down-regulated in the *in vivo* and *in vitro* PD models. However, up-regulating circDLGAP4 promoted cell viability and autophagy and inhibited apoptosis and mitochondrial damage, thereby alleviating the pathological changes of PD (Feng et al., 2020; Figure 2). These findings indicate that ncRNAs contribute to mitochondrial dysfunction by altering multiple mitochondrion-encoding genes, prompting the question of whether ncRNA-mediated mitochondrion-targeting therapeutics may be a viable treatment strategy for heterogeneous NDs.

## 3 TCM improves NDs by regulating mitochondrial dysfunction

Several clinal trials have been conducted to demonstrate the potential of NDs treatment using TCM. Approximately one-quarter of modern drugs are derived from natural products (Musthaba et al., 2010). Many TCMs exert neuroprotective effects to modulate mitochondrial dysfunction (Jiang et al., 2019). These effects include antioxidation, apoptosis inhibition, restoration of mitochondrial structure, biogenesis and dynamics, and mitophagy activation. The following section summarizes the TCM formulas with definite composition (Table 1), standardized extracts from individual TCMs (Table 2), and monomeric compounds isolated from TCM (Table 3) that can modulate mitochondrial dysfunction in ND-related models.

**TABLE 1:** List of traditional Chinese medicine (TCM) formulas treating neurodegenerative diseases (NDs) by alleviating mitochondrial dysfunction.

Disease	Formulations	Composition	*In-vitro*/*in-vivo* models/human trial	Mode of action	References
AD	Bushen Tiansui Recipe	*Asparagus cochinchinensis* (Lour.) Merr. (Tiandong), *Rehmannia glutinosa* (Gaertn.) DC. (Shudihuang), *Panax ginseng* C.A.Mey. (Renshen), *Phellodendron chinense* C.K.Schneid. (Huangbo), *Wurfbainia villosa* (Lour.) Škorničk. and A.D.Poulsen (Sharen), *Glycyrrhiza glabra* L. (Gancao), *Ligustrum lucidum* W.T.Aiton (Nüzhenzi), *Lycii Fructus* (Gouqizi), *Epimedium brevicornu* Maxim. (Yinyanghuo), *Conioselinum anthriscoides ‘Chuanxiong’* (Chuanxiong), *Hirudo* (Shuizhi)	Aβ_(1–42)_-induced AD rats	↑SOD, ↓MDA, ↓Mitochondrial swelling, ↑Antioxidation	Cao et al. (2019)
AD, PD	Dihuang Yinzi	*Polygala tenuifolia* Willd. (Yuanzhi), *Acorus calamus* var. *angustatus* Besser (Shichangpu), *Rehmannia glutinosa* (Gaertn.) DC. (Shudihuang), *Cornus officinalis* Siebold & Zucc. (Shanzhuyu), *Ophiopogon japonicus* (Thunb.) Ker Gawl. (Maidong), *Schisandra chinensis* (Turcz.) Baill. (Wuweizi), *Dendrobium nobile* Lindl. (Shihu), *Cistanche deserticola* Ma (Roucongrong), Gynochthodes officinalis (F.C.How) Razafim. & B.Bremer (Bajitian), *Aconitum carmichaelii* Debeaux (Fuzi), *Cinnamomum verum* J.Presl (Guangui), *Poria* (Fuling), *Zingiber officinale* Roscoe (Shengjiang), *Ziziphus jujuba* Mill. (Dazao), *Mentha canadensis* L. (Bohe)	Aβ_(1–42)_-induced AD rats; PD patients	↑PDH, ↑KGDH, ↓Mitochondrial swelling, ↑MMP, ↑Cognitive function	Huang et al. (2018), Zhu et al. (2021)
PD	Da-Bu-Yin-Wan (DBYW)	*Phellodendron chinense* C.K.Schneid. (Huangbo), *Anemarrhena asphodeloides* Bunge (Zhimu), *Rehmanniae Radix* Preaparata (Shudihuang), *Testudinia Crapax et Plastrum* (Guijia)	MPP^+^-treated SH-SY5Y cells	↑Mitochondrial mass, ↑Mitochondrial complex I activity, ↑Cellular ATP content	Zhang et al. (2016)
AD	Huangpu Tongqiao capsule	*Rheum officinale* Baill. (Dahuang), *Acorus calamus* var. *angustatus* Besser (Shichangpu), *Panax ginseng* C.A.Mey. (Renshen), *Conioselinum anthriscoides* ‘Chuanxiong’ (Chuanxiong), *Reynoutria multiflora* (Thunb.) Moldenke (Zhiheshouwu), *Alpinia oxyphylla* Miq. (Yizhi)	Aβ_(25–35)_-induced AD rats	↓Oxidative stress, ↓Mitochondrial apoptosis	Cai et al. (2018)
AD	Kaixin Powder	*Ginseng Radix et Rhizoma* (Renshen), *Poria* (Fuling), *Acorus calamus* var. *angustatus* Besser (Shichangpu), *Polygala tenuifolia* Willd. (Yuanzhi)	APP/PS1 transgenic mice	↓Mitochondrial damage, ↓Mitochondrial swelling, ↓Oxidativve stress	Xu et al. (2021)
AD	Sijunzi Decoction	*Ginseng Radix et Rhizoma* (Renshen), *Atractylodes macrocephala* Koidz. (Baizhu), *Poria* (Fuling), *Glycyrrhiza glabra* L. (Gancao)	D-Galactose (D-gal) induced AD rats	↑Mitochondrial complex CII and CIV activity, ↓AMPK	Liu et al. (2019a)
AD	Tianqi Yizhi granules	*Hedysarum polybotrys* Hand.-Mazz. (Hongqi), *Rhodiola crenulata* (Hook.f. and Thomson) H.Ohba (Hongjingtian)	Aβ_(1–42)_-induced AD rats	↑MMP, ↑Mitochondrial complex I, II, III, IV activity	Wu et al. (2015)
AD	Tongluo Xingnao effervescent tablet	*Conioselinum anthriscoides* ‘Chuanxiong’ (Chuanxiong), *Scutellaria baicalensis* Georgi (Huangqin), *Angelica sinensis* (Oliv.) Diels (Danggui)	APP/PS1 transgenic mice	↑MMP, ↑Energy charge levels, activity of respiratory chain complexes, ↑Na^+/^K^+^-ATPase activity	Yuan et al. (2016)
PD	Yinxing Pingchan Recipe	*Lonicera japonica* Thunb. (Jinyinhua), *Coptis chinensis* Franch. (Huanglian), *Astragalus mongholicus* Bunge (Huangqi), *Rehmannia glutinosa (Gaertn.)* DC. (Shudihuang), *Ginkgo biloba* L. (Yinxingye), *Pueraria montana* var. *lobata* (Willd.) Maesen and S.M.Almeida ex Sanjappa & Predeep (Gegen), *Rhodiola crenulata* (Hook.f. and Thomson) H.Ohba (Dayehongjingtian), *Paeonia lactiflora* Pall. (Baishao), *Gastrodia elata* Blume (Tianma), *Uncaria rhynchophylla* (Miq.) Miq. (Gouteng), *Glycyrrhiza glabra* L. (Gancao)	MPTP-induced PD mice	↑Mitochondrial enzyme complex activity, ↑Mitochondrial function	Sun et al. (2005)

**TABLE 2:** List of standardized extracts from individual traditional Chinese medicines (TCMs) exerting anti-neurodegenerative disease (anti-ND) effects by modulating mitochondrial dysfunction.

Disease	Single TCM extract	Family	*In-vitro*/*in-vivo* models/human trial	Mode of action	References
AD	20% ethanol extract of *Angelica sinensis* (Oliv.) Diels (Danggui)	Apiaceae	Aβ_(25–35)_ treated neuro 2A neuroblastoma cells	↓ROS, ↓TBARS, ↓GSH, ↑Neuroprotection, ↑MMP	Huang et al. (2008)
PD	30% ethanol extract of *Eleutherococcus senticosus* (Rupr. and Maxim.) Maxim. (Ciwujia)	Araliaceae	MPTP-induced PD mice	↑MMP, ↓Mitochondrial swelling	Liu et al. (2018)
ND	Water extract of *Coptis chinensis* Franch. (Huanglian)	Ranunculaceae	t-BOOH-induced SH-SY5Y cells	↓Apoptosis, ↑MMP	Friedemann et al. (2014)
ND	GBE	Ginkgoaceae	Aβ_(25–35)_, Aβ_(1–40)_, and Aβ _(1–42)_ induced hippocampal primary cultured cells; paraquat-induced PC12 cells	↑MMP, ↓Apoptosis, ↓RIP1-mediated mitochondrial dysfunction	Kang et al. (2007), Tu et al. (2020)
PD	GP	Araliaceae	PINK1^B9^ mutant drosophila melanogaster	↑Dopamine, ↑Mitochondrial unfolded protein response, ↑Mitochondrial function	Liu et al. (2020)
PD	Methanol extract of *Ganoderma lucidum* (Lingzhi)	Polyporaceae	MPTP-induced PD mice	↑Antioxidation, ↑Mitochondrial function, ↑Autophagy, ↓Apoptosis	Ren et al. (2019)
PD	70% ethanol extract of *Paeonia × suffruticosa* Andrews (Mudanpi)	Ranunculaceae	MPP-induced rat mesencephalic dopaminergic cells, MPTP-induced PD mice	↑Motor function, ↓Mitochondria-mediated apoptosis	Kim et al. (2014)
AD	Water extract of *Polygonum multiflorum* (Thunb.) Moldenke (Heshouwu)	Polygonaceae	Aβ_(1–40)_/Aβ_(25–35)_-induced rats	↑Mitochondrial COX activity, ↑Mitochondrial membrane fluidity	Um et al. (2006), Hou et al. (2008)
AD	RGE	Araliaceae	Aβ-induced HT22 Cells, 5×FAD mice	↑Mitochondrial dynamics, ↓Aβ deposits	Shin et al. (2019)

**TABLE 3:** List of monomeric compounds isolated from traditional Chinese medicines (TCMs) exerting anti-neurodegenerative disease (anti-ND) effects by regulating mitochondrial dysfunction.

Disease	Compound	Source	*In-vitro*/*in-vivo* models/clinical trials	Mode of action	References
AD, PD	Acteoside	*Cistanche deserticola* Ma (Roucongrong)	ICV-STZ induced AD rats; Aβ _(25–35)_-induced SH-SY5Y cells	↓ROS, ↑AMPK phosphorylation, ↓Mitochondria injury, ↑Autophagy	Aimaiti et al. (2021), Chen et al. (2021)
AD, PD	Andrographolide	*Andrographis paniculata* (Burm.f.) Nees (Chuanxinlian)	APP/PS1 transgenic mice; MPTP-induced PD mice	↓Oxidative stress, ↓Mitochondrial swelling, ↑Mitophagy	Geng et al. (2018), Geng et al. (2019)
AD, PD	Amentoflavone	*Selaginellae Herba* (Juanbai)	Aβ_(25–35)_-induced mice; MPTP-induced PD mice	↑ ratio of BCL2/BAX, ↑Autophagy	Cao et al. (2017), Cao et al. (2021)
AD, PD	Berberine and berberine derivative BBRP	*Coptis chinensis* Franch. (Huanglian)	Aβ_(1–42)_-induced primary cultured hippocampal neurons; MPTP-induced zebrafish	↓Toxicity, ↑MMP and ATP levels, ↓ROS, ↓MDA	Zhao et al. (2019), Wang et al. (2021b)
AD, PD	Cryptotanshinone	*Salvia miltiorrhiza* Bunge (Danshen)	PD-human-induced neuronal progenitor cells (hiNPCs); Aβ_42_-insulted SH-SY5Y cells	↓Cytotoxicity, ↓Apoptosis, ↑Mitochondrial restoration	Mei et al. (2012), Lee et al. (2020)
ND	Curcumin	*Curcuma longa* L. (Jianghuang)	hydroxynonenal-induced PC12 cells; tert-butyl hydroperoxide-induced AD rats	↓Apoptosis, ↑Antioxidation, ↓Mitochondrial dysfunction	Kumar and Singh (2015), Bagheri et al. (2020)
AD, PD	Crocin	*Crocus sativus* L. (Xihonghua)	L-glutamate-damaged HT22 cell; MPP^+^-induced P12 cells	↓ROS, ↓Cyt *c* release, ↓intracellular Ca^2+^, ↓mitochondrial dysfunction	Zhang et al. (2015a), Wang et al. (2019a)
PD	Celastrol	*Tripterygium wilfordii* Hook F. (Leigongteng)	MPP^+^-induced SH-SY5Y cells, MPTP-induced PD mice	↑Mitophagy (PINK1↑, DJ1↑, LRRK2↓), ↓Mitochondrial membrane depolarization, ↑Neuroprotection	Lin et al. (2019)
AD	Chikusetsusaponin V	*Panax notoginseng* (Burkill) F.H.Chen (Sanqi)	MPP-induced SH-SY5Y cells; H_2_O_2_-induced SH-SY5Y cells	↑MMP, ↓BCL2, ↑BAX, ↑BCL2/BAX ratio	Wang et al. (2021e)
AD, PD	Genistein	Many TCMs	Aβ_(25–35)_-induced cultured hippocampal neurons; intraperitoneal injection of D-galactose and intracerebral injection of Aβ _(25–35)_ to build an AD rat model; rotenone-induced SH-SY5Y cell	↓Mitochondrial apoptotic pathway, ↑Antioxidation, ↑Autophagy	Yan et al. (2016), Wu et al. (2018), Pierzynowska et al. (2019)
AD, PD	Geniposide	*Gardenia jasminoides* J.Ellis (Zhizi)	Oligomeric Aβ_(1–42)_-indeuced cortical neuron; APP/PS1 transgenic mice; MPTP-induced PD mice	↓Apoptosis, ↑Antioxidation, ↓ROS, ↓Mitochondrial dysfunction	Lv et al. (2014), Zhao et al. (2016), Chen et al. (2015)
AD	Huperzine A	*Huperzia serrata* (Thunb.) Trev. (Qiancengta)	Aβ_(25–35)_-insulted rat brain mitochondria; oligomeric Aβ_42_-induced primary rat neurons	↓Mitochondrial swelling, ↓ROS, ↑Mitochondrial respiration, ↑ATP synthesis	Gao et al. (2009), Lei et al. (2015)
PD	Isorhynchophylline	*Uncaria rhynchophylla* (Miq.) Miq. (Gouteng)	MPP-induced PC12 cells	↑ASK1/JNK signaling-mediated mitochondria-dependent apoptosis pathway	Li et al. (2017b)
PD	Kukoamine A	*Lycii Cortex* (Digupi)	6-OHDA-induced PD model of PC12 cells	↓Mitochondrial apoptosis, ↑MMP, ↓ROS, ↓MDA, ↑SOD	Li et al. (2021b)
ND	Morroniside	*Cornus officinalis* Siebold & Zucc. (Shanzhuyu)	H_2_O_2_-induced SK-N-SH human neuroblastoma cells	↑Antioxidation, ↓Apoptosis, ↓JNK and p38 MAPK phosphorylation	Zhang et al. (2017)
AD	Notoginsenoside R1	*Panax notoginseng* (Burkill) F.H.Chen (Sanqi)	PC12 neuronal cells incubated with Aβ _(25–35)_	↑Antioxidation, ↓Apoptosis, ↑MMP, ↓MAPK signaling	Ma et al. (2014)
AD, PD, ALS	Oxymatrine	*Sophora flavescens* Aiton (Kushen)	Aβ_(1–42)_-induced primary neuronal cells; MPTP-induced mice, MPP^+^-induced mice primary microglia; SOD1-G93A transgenic mice	↑BCL2/BAX, ↓caspase-3, ↓HMGB1/TLR4/NF-κB signaling	Dong et al. (2019), Gan et al. (2020), Zhang et al. (2021a)
PD	Paeoniflorin	*Paeonia lactiflora* Pall. (Baishao)	Rotenone-induced PC12 cells	↑MMP, ↓Apoptosis	Liu et al. (2016)
AD, PD	Quercetin	Many TCMs	6-OHDA-treated PC12 cells; streptozotocin (STZ)-induced AD rats	↑Cell viability, ↓Mitochondrial damage, ↑α7nAChR/Nrf2/HO-1-mediated neuroprotection	Wang et al. (2021d), Singh and Garabadu (2021)
AD, PD, HD	Resveratrol	*Reynoutria japonica* Houtt. (Huzhang)	APP/PS1 transgenic mice; rotenone-induced PC12 cells; YAC128 transgenic mice embryos, HD human lymphoblasts	↓ROS, ↑Autophagy, ↑Mitochondria dynamics, ↑MMP	Peng et al. (2016), Naia et al. (2017), Han et al. (2020)
ND	Salidroside	*Rhodiola crenulata* (Hook.f. and Thomson) H.Ohba (Hongjingtian)	Aβ_(1–42)_-induced PC12 cells; MPP^+^-injured SN4741 cells, MPTP-lesioned mice	↓Apoptosis, ↑Mitochondrial MEF2D-ND6 pathway, ↑ERK1/2 and AKT signaling pathways	Liao et al. (2019), Li et al. (2020)
AD, PD	Silibinin (Silymarin)	*Silybum marianum* (L.) Gaertn. (Shuifeiji)	PC12APPsw cells; MPTP-induced PD mice	↓ROS, ↑MMP, ↓Mitochondrial fission, ↑Mitochondrial fusion, ↑Mitophagy	Liu et al. (2021b), Liu et al. (2021c), Esselun et al. (2021)
AD	Triptolide	*Tripterygium wilfordii* Hook F. (Leigongteng)	Aβ_(25–35)_-induced PC12 cells	↓Intracellular Ca^2+^, ↓Apoptosis	Xu et al. (2016)
AD, PD	*β*-asarone	*Acorus calamus* var. *angustatus* Besser (Shichangpu)	APP/PS1 transgenic mice, Aβ_(1–42)_-induced PC12 cells; unilateral medial forebrain bundle lesion PD rats	↑Mitochondrial autophagy	Huang et al. (2017), Wang et al. (2021c)
AD	Vitegnoside	*Vitex negundo* L. (Huangjin)	copper-treated SH-SY5Y cell line carrying the Swedish mutation	↑MMP, ↓Cyt *c* release, ↓BAX/BCL2 ratio, ↓caspase-9/-3 activity	Wang et al. (2019b)
PD	2,3,5,4’-tetrahydroxystilbene-2-O-β-d-glucoside (TSG)	*Reynoutria multiflora* (Thunb.) Moldenke (Heshouwu)	MPTP-induced PD mice	↓ROS, ↓Mitochondria-mediated apoptosis	He et al. (2015)

### 3.1 TCM formulas with a definite composition

Many classical formulas documented in ancient Chinese medical books can exert neuroprotective effects, and the efficacy of these formulas in treating NDs has been demonstrated by several studies using modern testing techniques. The compatibility of TCM formulas ensures a synergistic effect by increasing the dissolution rate of the major active compounds while reducing the content of toxic components in TCM. This provides a good safety profile of the compounds while exerting therapeutic effects. One study summarized the drug use pattern of TCM formulas for AD treatment and found that 150 TCMs were used with 132 formulas, among which *Acorus calamus* var. *angustatus* Besser (Shichangpu) was the most frequently used TCM (Hu et al., 2012; Ma et al., 2015).

Dihuang Yinzi, consisting of twelve Chinese herbs, improved the daily living and cognitive activities in patients with mild to moderate AD in clinical trials, and its efficacy might be superior to that of donepezil (Zhang et al., 2018). The medicinal chemistry of the cerebrospinal fluid was evaluated after administering Dihuang Yinzi, and five migrating components were found in the cerebrospinal fluid. Among the five components, one was the prototype component contained in Dihuang Yinzi [schizandrin derived from *Schisandra chinensis* (Turcz.) Baill. (Wuweizi)] and four were novel metabolites produced by *Schisandra chinensis* (Turcz.) Baill. (Wuweizi), *Polygala tenuifolia* Willd. (Yuanzhi), *Ophiopogon japonicus* (Thunb.) Ker Gawl. (Maidong), and *Gynochthodes officinalis* (F.C.How) Razafim. and B. Bremer (Bajitian). The four novel components might be the main pharmacologically active compounds that prevent cognitive dysfunction (Guo, 2016). Recent studies showed that Dihuang Yinzi significantly increased the PDH and KGDH levels, the key enzymes of the mitochondrial tricarboxylic acid cycle, in the brain of AD rats. Thus, Dihuang Yinzi promoted the tricarboxylic acid cycle and improved glucose utilization. It also significantly increased the mitochondrial membrane potential (MMP), protected the integrity of the inner mitochondrial membrane, and reduced mitochondrial swelling by depolarizing the inner mitochondrial membrane (Huang et al., 2018).

Relevant research has found that the water extract of Sijunzi decoction facilitates Aβ transportation across the blood-brain barrier (BBB) and reduces the aggregation of Aβ plaques in the brain (Guo et al., 2020). Further analysis of the pharmacological ingredients of Sijunzi decoction revealed that ginsenoside Rh2, panaxadiol, *Poria cocos* polysaccharide, and isoliquiritigenin were the principal active ingredients of the decoction (Zhang et al., 2021b). Furthermore, recent studies showed that Sijunzi decoction promotes central energy generation and minimizes behavioral abnormalities in AD by modulating the activities of respiratory complex II and III of the mitochondrial electron transport chain and AMP-dependent protein kinase (AMPK) signaling pathway (Liu et al., 2019a). AMPK first activates factors related to the mitochondrial quantity and quality regulation to provide mitochondrial protection. Recent studies reported that AMPK inhibits the expression of ATP synthase inhibitor protein, suggesting that AMPK inhibition may stimulate the oxidative respiratory chain and promote energy production. Moreover, the AMPK content was reduced in the groups treated with Sijunzi decoction, indicating that Sijunzi decoction might effectively treat AD by stimulating the mitochondrion-dominated central energy production.

Yinxing Pingchan recipe, composed of eleven herbal medicines, is a TCM product with years of clinical application in PD therapy. This recipe can significantly modulate the antioxidant system of cells, increasing their ability to resist free radical damage and effectively reduce MDA production (Zhang et al., 2004). The dismantled formulas of Yinxing Pingchan recipe (such as *Ginkgo biloba* L. (Yinxingye), *Pueraria montana* var. *lobata* (Willd.) Maesen and S.M.Almeida ex Sanjappa & Predeep (Gegen), and *Rhodiola crenulata* (Hook.f. and Thomson) H.Ohba (Dayehongjingtian)) also have significant antioxidant effects, which reduce free radicals and lipid peroxide levels. These effects have been demonstrated to be associated with the ability to enhance the survival of DA neurons. Additionally, these dismantled formulas inhibited MPTP-induced DA neuron loss and apoptosis to varying degrees in the substantia nigra of the PD mouse model. Thus, it is speculated that the Yinxing Pingchan recipe may protect the mitochondria and prevent apoptosis of DA neurons by enhancing mitochondrial enzyme complex activity, eventually slowing down the progression of PD (Sun et al., 2005).

### 3.2 Standardized extracts from individual TCMs

Several preclinical and human trials suggested that standardized extracts from individual TCMs may have anti-AD activities. *Ginseng Radix et Rhizoma Rubra* (Hongshen) and *Ginseng Radix et Rhizoma* (Renshen) are from the same family and have similar pharmacological effects due to their partially identical compounds. The water extract of *Talinum paniculatum* (Jacq.) Gaertn. (RGE) significantly alleviated Aβ-induced mitochondrial pathology by reducing the mitochondrial fusion/fission imbalances and restoring damaged mitochondrial respiratory chains in the *in vivo* and *in vitro* AD models. This suggested that RGE may be a mitochondria-targeted medicine for treating AD (Shin et al., 2019).

*Eleutherococcus senticosus* (Rupr. and Maxim.) Maxim. (Ciwujia) extract (EAS) demonstrated extensive therapeutic effects *via* EAS-enhanced motor coordination in a PD mouse model. Analysis at the superstructural level revealed that EAS prevented diencephalon mitochondrial swelling and attenuated the decrease of MMP. EAS also inhibited oxidative stress and restored the normal expression of PD-related proteins [Parkin, PINK1, DJ1, α-syn, and leucine-rich repeat kinase 2 (LRRK2)], demonstrating that EAS exerts its neuroprotective effects in PD by ameliorating mitochondrial dysfunction and structural damage (Liu et al., 2018). Modern pharmacological studies showed that the active compounds of *Ginseng Radix et Rhizoma* (Renshen) have a protective effect against neurotoxicity in different PD models. Ginseng protein (GP) is an important pharmacologically active compound that has been shown to exhibit neuroprotective effects in AD (Li et al., 2017a). It was found that GP treatment delayed the onset of PD-like phenotypes, promoted mitochondrial function, and protected mitochondria from oxidative stress-induced damage (Liu et al., 2020). This further confirmed the potential and therapeutic mechanisms of *Ginseng Radix et Rhizoma* (Renshen) as a valuable PD treatment.

*Ginkgo biloba* extract (GBE), a mixture obtained from *Ginkgo biloba* L. (Yinxingye), has a unique pharmacological activity making it one of the most commonly used drugs in the clinical prevention and treatment of AD. GBE contains two classes of pharmacologically active components; terpenes (including bilobalide and ginkgolide A, B, and C) and flavonoids (including meletin, isorhamnetin, and kaempferol). GBE has been demonstrated to directly regulate mitochondria through multiple mechanisms in various *in vivo* and *in vitro* AD models. For example, GBE improves mitochondrial function by increasing MMP and ATP levels or stimulating mitochondrial biogenesis by improving the damaged mitochondrial respiratory chain (Stockburger et al., 2018). EGb761 contains 24% of flavonoid glycosides and 6% of terpenes (ginkgolide A, B, and C, 2.8%–3.4%; bilobalide, 2.6%–3.2%), and the ginkgolide and bilobalide showed significant mitochondrial protective characteristics (Lejri et al., 2019). Furthermore, *in vivo* and *in vitro* experiments showed that treating AD with EGb761 significantly increased the viability of mitochondrial complex I, IV, and V and reversed MMP and ATP production in a dose-dependent manner. A clinical trial indicated that EGb761 protected patients from neurological dysfunction and improved their cognitive ability by inhibiting the damaging effects of oxidative free radicals on neurons (Zhu et al., 2017). GBE also has efficacy in treating other NDs; for example, EGb761 protected against MPTP-induced neuronal or neuron-like cell apoptosis in PD by increasing the activation of BCL2, maintaining the stability of MMP, and reducing the activation of caspase-3 through a mitochondrion-dependent pathway. This indicated the potential of EGb761 therapy in treating AD (Kang et al., 2007). Additionally, *in vivo* and *in vitro* preclinical studies and relevant clinical trials suggest that EGb761 may have promising therapeutic effects for preventing and treating AD and other age-related NDs (Singh et al., 2019).

### 3.3 Monomeric compounds isolated from TCMs

TCMs exert their curative effects through the isolated active compounds. Many TCMs have been pharmacodynamically characterized, and some, such as *Andrographis paniculata* (Burm.f.) Nees (Chuanxinlian), *Crocus sativus* L. (Xihuanghua), *Gardenia jasminoides* J.Ellis (Zhizi), *Tripterygium wilfordii* Hook F. (Leigongteng), have been shown to effectively improve damaged neurological function.

Andrographolide (AG), a diterpene lactone compound, is one of the main active ingredients of *Andrographis paniculata* (Burm.f.) Nees (Chuanxinlian). With the recent continuous research on the pharmacological functions of AG, several studies have shown that AG has good neuroprotective effects and promising clinical applications (Lu et al., 2019). A seven-month-long prophylactic administration of AG sulfonate reduced the oxidative stress and mitochondrial swelling in the Aβ precursor protein (APP)/Presenilin-1 (PS1) transgenic mice (Geng et al., 2018). AG also prevented excessive mitochondrial fission and neuronal damage in the striatum of PD mice by binding to DRP1, a target AG protein (Geng et al., 2019).

Crocin, an ester compound with antioxidant and anti-inflammatory effects from *Crocus sativus* L. (Xihonghua), reportedly delays the development of neurological diseases (Farkhondeh et al., 2018). Crocin pretreatment in an *in vitro* AD model significantly increased cell viability, reduced apoptosis, alleviated mitochondrial dysfunction, and inhibited intracellular oxidative stress and Ca^2+^ overload (Wang et al., 2019a). Furthermore, crocin also inhibited mitochondrial dysfunction in PD by restoring MMP and ATP synthesis and inhibiting the cytochrome *c* (Cyt *c*) release into the cytoplasm (Zhang et al., 2015a). In addition to *Crocus sativus* L. (Xihuanghua), crocin is also one of the active compounds of *Gardenia jasminoides* J.Ellis (Zhizi). However, the main pharmacologically active ingredient of *G. jasminoides* J.Ellis (Zhizi) is geniposide, which exhibits similar pharmacological effects as crocin. Research has proven that geniposide is protective against mitochondrial dysfunction by preventing AD progression (Lv et al., 2014; Zhao et al., 2016). Moreover, geniposide also exerted neuroprotective effects in PD through the mitochondrion-mediated apoptotic pathway (Chen et al., 2015). Among many anti-free radical natural products, silibinin is one of the few drugs widely used in clinical practice. The exact efficacy and low toxicity of silibinin were demonstrated through clinical applications over the last 30 years. Recent studies reported that silibinin alleviated motor dysfunction in a PD mouse model by inhibiting oxidative stress, neuroinflammation, and imbalance of mitochondrial dynamics and promoting mitophagy and mitochondrial biogenesis (Liu et al., 2021b; Liu et al., 2021c). In an *in vivo* AD model, the silibinin intervention significantly reduced the Ca^2+^ overload-induced mitochondrial swelling and improved the fluidity of the mitochondrial membrane (Esselun et al., 2021).

Huperzine A (HupA) is a sesquiterpenoid alkaloid isolated from *Huperzia serrata* (Thunb.) Trev. (Qiancengta), whose potential in treating AD has been demonstrated through numerous preclinical studies and clinical trials (Yang et al., 2013). HupA exhibited a good safety profile in clinical trials without serious adverse events. It has been proposed that the neuroprotective mechanism of HupA involves improving energy metabolism and preserving the mitochondrial structure. This was evidenced by the inhibition of Aβ-induced decrease in mitochondrial respiration, ATP synthesis, and transmembrane potential, and the effective prevention of Cyt *c* release, ROS increase, and mitochondrial swelling in the *in vivo* and *in vitro* experiments (Gao et al., 2009; Lei et al., 2015). Early studies suggested that salidroside, the main active compound of *Rhodiola crenulata* (Hook.f. and Thomson) H.Ohba (Hongjingtian), is a potential neuroprotective agent (Zhong et al., 2018). Salidroside improved cell viability by inhibiting Aβ_1-42_-induced cytotoxicity and the mitochondrion-mediated endogenous apoptotic pathway (Liao et al., 2019). Damaged mitochondrial complex I and oxidative stress play a crucial role in degenerating dopaminergic (DA) neurons during PD progression. Studies showed that treating PD with salidroside improved cell viability, inhibited apoptosis, restored MMP and Mitochondrial Complex I activity, and protected DA neurons by mediating the mitochondrial MEF2D-ND6 pathway (Li et al., 2020). Triptolide (a diterpenoid) and celastrol (a triterpenoid) are the most active components of *Tripterygium wilfordii* Hook F. (Leigongteng), which have been used to effectively treat several rheumatic diseases and immune system disorders (Tong et al., 2021). Several studies demonstrated that triptolide and celastrol have protective effects on neurons and glial cells but through different mechanisms. The apoptosis and oxidative stress inhibition is the key protective mechanism of triptolide against AD (Xu et al., 2016). Unlike triptolide, celastrol ensures mitochondrial quality by isolating damaged mitochondria for autophagosome degradation. This suggests that celastrol reduces DA neuronal death, mitochondrial membrane depolarization, and ATP reduction by activating mitochondrial autophagy to degrade damaged mitochondria, and inhibiting apoptosis of DA neurons, providing a new prevention and treatment mechanism for PD (Lin et al., 2019).

## 4 TCM prevents mitochondrial dysfunction in NDs by regulating ncRNAs

Currently, the active compounds of TCM reportedly regulate the expression of genes through ncRNAs, which participate in various signaling pathways associated with different therapeutic activities, such as anti-tumor, anti-inflammation, and anti-atherosclerosis activities. However, there are fewer reports on anti-ND research, especially mitochondrial dysfunction modulation for treating NDs, which is still in its early stages. The TCMs regulating various mitochondrial dysfunctions by targeting ncRNAs associated with NDs are listed in Table 4. The subsequent section summarizes several active compounds of TCM that have been well-studied.

**TABLE 4:** Summary of traditional Chinese medicines (TCMs) alleviating mitochondrial dysfunction in neurodegenerative diseases (NDs) by modulating non-coding RNAs (ncRNAs).

Disease	TCM	ncRNAs	*In-vitro*/*in-vivo* models/human trial	Mode of action	References
PD	*Eleutherococcus senticosus* (Rupr. and Maxim.) Maxim. (ciwujia)	miR-205, miR-433, miR-153	MPP^+^-induced PC12 cells	↓Mitochondrial swelling, ↑MMP, ↓ROS	Liu et al. (2018), He et al. (2019)
AD	Ampelopsin	miR-34a	D-gal-induced rats	↓Apoptosis, ↓Astrocyte activation, ↑Autophagy	Kou et al. (2016)
AD	Berberine	lncBACE1-AS/miR-132-3p, circHDAC9/miR-142-5p, LINC00943/miR-142-5p, miR-188	Aβ_(25–35)_-induced HPN and SK-N-SH cells; Aβ_42_-induced human neuronal cells; Aβ-treated BV2 and N2a cells	↑Neuroprotection, ↓ROS, ↓Apoptosis	Chen et al. (2020), Ge et al. (2020), Zhang et al. (2020), Li et al. (2021a)
AD	Curcumin	miR-146a, miR-125b, miR-15b-5p	magnesium-, iron-, gallium-, and aluminum-sulfate-stressed human-astroglial (HAG) cells; 5 different transgenic mouse models; swAPP695-HEK293 cells	↓ROS, ↑Autophagy, ↓Neuroinflammation	Li et al. (2011), Pogue et al. (2011), Liu et al. (2019b), Gong and Sun (2020)
AD	Ginsenoside Rg1 and *Acorus calamus* var. *angustatus* Besser (Shichangpu) extract	miR-873-5p	SAMP1 and SAMP8 mice	↓Apoptosis	Shi et al. (2018)
AD, PD	Resveratrol	miR-134, miR-214, MALAT1/miR-129	MPTP-induced PD mice, MPP^+^-induced SH-SY5Y cells; APP/PS1 transgenic mice	↑Antioxidation, ↓Apoptosis, ↑Neuroprotection	Zhao et al. (2013), Wang et al. (2015), Shen et al. (2018), Xia et al. (2019)
PD	Tanshinone IIA	miR-153	6-hydroxydopamine (6-OHDA)-treated SH-SY5Y cells	↑MMP, ↓Cyt *c* translocation	Zhang et al. (2015b)

Curcumin is a polyphenol and a principal bioactive component of *Curcuma longa* L. (Jianghuang), whose neuroprotective effects have been well-studied because of its pleiotropy and broad-spectrum targets in the brain. Several studies reported that curcumin maintains mitochondrial dynamics, biogenesis, synaptic transmission, and integrity by improving mitochondrial fusion/fission balance (Reddy et al., 2016). Moreover, curcumin exhibits better effects when used with other compounds, such as quercetin (Waseem and Parvez, 2016) and berberine (Lin et al., 2020). Various curcumin derivatives improve its bioavailability, stability, and BBB permeability and enhance its neuroprotective and mitochondrial-protective effects (Bagheri et al., 2020). Further research investigated whether curcumin treatment of NDs is associated with ncRNAs. The results showed that during AD development, miR-125b, miR-146a, and miR-15b-5p were significantly up-regulated (Li et al., 2011). A recent study found that curcumin, an NF-κB inhibitor, significantly reduced miR-146a and miR-125b levels (Pogue et al., 2011) by up-regulating the expression of the complement factor H protein, inhibiting M1 microglia phenotype, reducing the inflammatory response, and promoting phagocytosis and clearance of Aβ plaques (Gong and Sun, 2020). Another study showed that curcumin up-regulated miR-15b-5p expression to reduce APP and Aβ levels in swAPP695-HEK293 cells (Liu et al., 2019b). Furthermore, curcumin modulated oxidative stress, apoptosis, and neuroinflammation-mediated mitochondrial dysfunction by targeting miRNAs, indicating its potential development as a drug for treating NDs (Xu et al., 2019).

The role of berberine (isoquinoline alkaloid), the main active ingredient extracted from *Coptis chinensis* Franch. (Huanglian), in neurological disorders has been increasingly explored. In recent years, berberine has been developed to protect motor neurons and exhibits potential therapeutic effects in neurodegenerative lesions. Additionally, some studies have confirmed the role of berberine in maintaining synaptic structure and function. Berberine improved mitochondrial dynamics and biogenesis and prevented synaptic loss by maintaining the MMP and preventing ATP reduction (Zhao et al., 2019). Although the underlying protective mechanisms of berberine remain elusive, results demonstrated the berberine derivative, BBRP, rapidly and specifically accumulated in mitochondria and inhibited the accumulation of PINK1 protein when it exerted anti-PD effects. This suggested that the potential berberine target in the brain for PD therapy is mitochondria (Wang et al., 2021b). It has been reported that berberine could inhibit the NF-κB pathway by down-regulating LINC00943 and up-regulating miR-142-5p levels, thereby protecting the cells from MPP-induced neuronal injury (Li et al., 2021a). Furthermore, berberine elevated circRNA HDAC9 expression and reduced miR-142-5p level, attenuating the toxicity in human nerve cells in AD (Zhang et al., 2020). Another study showed that berberine inhibited apoptosis and oxidative stress but activated synaptic activity and plasticity by acting on lncRNA BACE1-AS to up-regulate miR-132-3p level in neurons, inducing a significant restoration of Aβ-induced neuronal cell viability (Ge et al., 2020). Moreover, it was shown that berberine accelerated cell viability and inhibited caspase-3 activity and apoptosis through the miR-188/NOS1 axis, thereby reducing neuronal injury (Chen et al., 2020). Thus, based on these studies, berberine exerts its neuroprotective effects by targeting multiple miRNAs, lncRNAs, circRNAs, and various signaling pathways, providing new targets for the treatment action of NDs.

Resveratrol, a natural polyphenol, is one of the active ingredients of *Reynoutria japonica* Houtt. (Huzhang) and is found in various natural plants. Numerous *in vivo* and *ex vivo* experiments showed that resveratrol exerts neuroprotective effects in various NDs, including AD, PD, HD, and ALS. Moreover, resveratrol regulates mitochondrial dysfunction in NDs through different mechanisms. In AD, resveratrol prevented Aβ-induced mitochondrial fusion/fission imbalance by maintaining the levels of dynamics-related genes (MFN2 and DRP1). Resveratrol also inhibited the activity of Cyt *c* oxidase, alleviating mitochondrial swelling and fragmentation (Yang et al., 2020). In addition to the aforementioned molecular mechanisms, resveratrol might also act by down-regulating miR-134 and miR-124 expression, stimulating the CREB/BDNF signaling pathway and restoring synaptic plasticity to improve learning and memory (Zhao et al., 2013; Shen et al., 2018). In PD, resveratrol administration modulated mitochondrial biogenesis by enhancing mitochondrial mass and fusion/fission balancing, increasing the ATP levels (Peng et al., 2016). Moreover, the level of miR-214 was significantly reduced in the mesencephalon of PD mice and MPP^+^-induced SH-SY5Y cells; however, treatment with resveratrol increased the miR-214 expression level in the PD-associated models and reduced mRNA and protein expression of α-syn (Wang et al., 2015). Recently, miR-214 has been suggested to target genes regulating neuronal growth and differentiation in various ND models. Furthermore, resveratrol administration ameliorated PD symptoms by modulating the MALAT1/miR-129/SNCA pathway involved in mitochondrion-mediated apoptosis (Xia et al., 2019), suggesting a potential association of resveratrol action with miRNA function and mitochondrial signal.

## 5 Summary and prospect

Because of their diverse phenotypes and complex pathogenesis, there have been no effective drugs to cure NDs. However, due to their therapeutic effects, TCMs may act as potential ncRNA regulators, providing a new perspective for elucidating the “multi-compound-multi-target-multi-pathway” treatment mechanism of NDs. Mitochondrial dysfunction is an early pathological phenomenon in NDs, important for drug discovery in preclinical studies. This review explored the mechanisms by which the most studied ncRNAs contributed to the mitochondrial dysfunction in NDs. The review also focused on the TCM formulas (including Sijunzi Decoction and Dihuang Yinzi), standardized extracts from individual TCMs [e. g. *Eleutherococcus senticosus* (Rupr. and Maxim.) Maxim. (Ciwujia) extract and GBE] and the monomeric compounds isolated from various TCMs (such as andrographolide, crocin, and silibinin) with promising clinical applications in treating NDs. These active components of TCM formulas exerted neuroprotective effects in various ND models by targeting mitochondria. The neuroprotective effects included inhibiting oxidative stress, suppressing apoptosis, activating mitophagy, and maintaining mitochondrial homeostasis. Furthermore, we reviewed the ongoing research efforts on representative components which regulate ncRNA-evoked mitochondrial function and pathways for NDs therapy. Given the complexity and instability of the TCM ingredients, further analysis and exploration of the multi-targets and multi-pathways involved in the ncRNA-mitochondrial regulation network are needed. Therefore, understanding the role and mechanisms of ncRNAs in mitochondrial function will help discover new targets and potential drug candidates for NDs.
